# The Summary Index of Malaria Surveillance (SIMS): a stable index of malaria within India

**DOI:** 10.1186/1478-7954-8-1

**Published:** 2010-02-11

**Authors:** Alan A Cohen, Neeraj Dhingra, Raju M Jotkar, Peter S Rodriguez, Vinod P Sharma, Prabhat Jha

**Affiliations:** 1 Centre for Global Health Research, Li Ka Shing Knowledge Institute, St Michael's Hospital, Toronto, Ontario, Canada; 2 National AIDS Control Organization, Delhi, India; 3 Centre for Global Health Research, St John's Research Institute, Bangalore, Karnataka, India; 4 National Institute of Malaria Research, Delhi, India

## Abstract

**Background:**

Malaria in India has been difficult to measure. Mortality and morbidity are not comprehensively reported, impeding efforts to track changes in disease burden. However, a set of blood measures has been collected regularly by the National Malaria Control Program in most districts since 1958.

**Methods:**

Here, we use principal components analysis to combine these measures into a single index, the Summary Index of Malaria Surveillance (SIMS), and then test its temporal and geographic stability using subsets of the data.

**Results:**

The SIMS correlates positively with all its individual components and with external measures of mortality and morbidity. It is highly consistent and stable over time (1995-2005) and regions of India. It includes measures of both *vivax *and *falciparum *malaria, with *vivax *dominant at lower transmission levels and *falciparum *dominant at higher transmission levels, perhaps due to ecological specialization of the species.

**Conclusions:**

This measure should provide a useful tool for researchers looking to summarize geographic or temporal trends in malaria in India, and can be readily applied by administrators with no mathematical or scientific background. We include a spreadsheet that allows simple calculation of the index for researchers and local administrators. Similar principles are likely applicable worldwide, though further validation is needed before using the SIMS outside India.

## Background

Malaria in India has a long and tumultuous history. Apparently not widespread before British agricultural projects created ideal breeding conditions for the mosquito vectors, by the end of the 19^th ^century, malaria had become a severe public health concern: a constant endemic problem in northeastern regions such as Bengal and a periodically ravaging epidemic in the northwestern states such as Punjab, where a single epidemic killed in excess of 300,000 people in late 1908 [1-3]. During this time, *falciparum *malaria - substantially more severe and deadly than the other species - became widespread. After independence, a control program nearly succeeded in eliminating malaria entirely, but in 1965, on the verge of success, funding was cut, and there was a substantial rebound of the disease in the following years[4,5]. Currently, malaria is much less severe than before the control program, but it continues to be a major public health concern, accounting for perhaps 1-2% of all deaths in India (AAC and PJ, unpublished data). In some states, particularly Orissa, disease burden is much worse[5].

As part of the National Malaria Eradication Programme (which became the National Vector-born Disease Control Program, or NVBDCP), a surveillance system was set up in 1958 to measure malaria incidence based on examination of blood smears at Primary Health Centers (PHCs)[1]. However, because most of the surveillance is passive, slides are much more likely to come from people who have malaria than expected from a random sample of the population. These measures are thus not a reliable way to estimate overall incidence, morbidity, or mortality. Better anti-malarial treatment and surveillance in high-malaria areas may also result in relative underestimation of malaria in low-malaria areas.

Statistics are compiled yearly for each district. Convention and the nature of the data collection have resulted in the calculation of seven different indices for each district in each year (see Methods for details), each with a slightly different interpretation. Some measures are for all malaria; others just for the more severe species, *falciparum*. This has resulted in the need to present many different graphs or columns to show trends for so many indices, and it is not always clear how to interpret countervailing trends in different indices[5]. Further, each index has strengths and weaknesses, and none alone seems to adequately summarize malaria levels for an area.

Here, using principal components analysis (PCA), we combine the existing measures into the Summary Index of Malaria Surveillance (SIMS), a single summary index of malaria trends. This index is scaled between 0 and 100, with higher numbers indicating more malaria, making it easy for laymen to interpret. We confirm the validity of this index using both internal and external validation. Internal validation includes confirming that (a) all measures load in the same direction on the index (the first PCA axis); (b) the first PCA axis explains a substantial portion of the variation; (c) the axis is robust when generated from different subsets of the data; and (d) the axis is robust when generated from different combinations of the measures. External validation is conducted by assessing the correlation of our index and the original measures with independent measures of malaria mortality and morbidity in India from the Million Death Study (MDS) and District-level Household Survey (DLHS), respectively. Lastly, we provide a Microsoft Excel spreadsheet that can easily be used by researchers and local officials to calculate the SIMS from raw data.

## Methods

### Data

#### NVBDCP (National Vector-born Disease Control Program)

The NVBDCP in India collects laboratory surveillance data (peripheral blood smears) primarily from patients who present themselves with fevers at a Primary Health Center (PHC) or any public health facility, instructing health workers to take blood smears (passive surveillance). In addition, health workers visit households in their jurisdictions once each fortnight and ask if anybody is presently suffering from fever or has since the last visit. If yes, a blood smear is collected, and chloroquine tablets are given as a presumptive treatment (active surveillance). The slides are then examined for evidence of malaria, and this is recorded as being negative, positive for *Plasmodium falciparum*, positive for other *Plasmodium *species (*vivax *or *malariae*, mostly *vivax*), or positive for both *P. falciparum *and other species. On receipt of positive results, radical treatment for malaria is supposed to be given to the patient. This should happen before development of gametocytes in the body (within 21 days) to halt the transmission (Table 1)[5] (GPS Dhillon and GS Sonal, personal communication). Time lag between collection of the slide and administration of treatment is an operational quality indicator for the program.

**Table 1:** Current protocol for malaria surveillance and treatment in India

Level	Government Strategy/Activities	Private sector response
Household/where there is no doctor	• Active surveillance of fever cases (home visit of HW) • Presumptive treatment with chloroquin • Peripheral blood smear • If the result is positive then radical treatment with Primaquin for appropriate duration based on whether it is Pv or Pf.	• Over-the-counter incomplete treatment by pharmacists

PHC or health facility (doctor available)	• Passive surveillance of fever cases (attendees of the facility) • If there is no facility for blood smear examination then presumptive treatment with chloroquin, with a peripheral blood smear or rapid test for Pf taken for subsequent analysis. If the result is positive then radical treatment with Primaquin for appropriate duration based on whether it is Pv or Pf. • If there is a facility for blood smear examination (malaria clinic), peripheral blood smear and decide course of treatment based on the results (PT/PT +RT/Post RT/IPT).	• Case management based on clinical impression, Peripheral Blood smear/Rapid test for Pf/Quantitative Buffy coat/Indirect tests to detect Malaria. • Use of Mefloquin/ACT

Referral hospital (specialist doctor available)	• Case management of walk-in as well as referred malaria fever based on clinical impression, peripheral blood smear, rapid test for Pf, quantitative buffy coat, and indirect tests to detect malaria. • Decide course of treatment based on the results (PT/PT +RT/Post RT/IPT) • Use of Mefloquin/ACT is common	• Case management of walk-in as well as referred malaria fever based on clinical impression, peripheral blood smear, rapid test for Pf, quantitative buffy coat, and indirect tests to detect malaria. • Use of Mefloquin/ACT

These results are then compiled at the district level - there are currently about 600 districts in the 35 Indian states and union territories - and used to generate a series of statistics. The raw numbers collected include: population of the district in thousands ("Pop"); blood smears collected ("BSC"); blood smears examined ("BSE"); # of slides positive for *P. vivax *or *malariae *("Pv"); # of slides positive for *P. falciparum *("Pf"); and # of slides positive for both ("mixed"). These raw numbers are then used to calculate several indices: total number of positive slides ("positive" = Pv + Pf + mixed); percent of positive slides that are positive for *P. falciparum *("%Pf" = (Pf + mixed)/positive); annual blood examination rate ("ABER" = BSE/Pop/10); annual parasite index ("API" = positive/Pop); annual *falciparum *index ("AFI" = (Pf + mixed)/Pop); slide positivity rate ("SPR" = 100 × positive/BSE); and slide *falciparum *rate ("SFR" = 100 × (Pf + mixed)/BSE). The number of malaria deaths certified by the NVBDCP ("deaths") is also recorded. The measures traditionally used to monitor malaria levels are %Pf, ABER, API, AFI, SPR, SFR, and deaths.

Each of the measures above has a particular interpretation. ABER measures coverage of the surveillance program, and potentially also local fever incidence. Convention suggests that when ABER is less than 10%, coverage is poor enough that population-referent measures such as API and AFI should be viewed with skepticism[5]. However, ABER may be associated with malaria rates to the extent that sick people seek treatment and therefore have slides examined. *Falciparum*-positivity is important to distinguish from overall positivity because *falciparum *malaria is more deadly. API and AFI, though not true measures of population prevalence or incidence, do provide an approximation of disease burden in the population, because presumably many who fall ill do come to PHCs or health facilities for treatment and thus have slides taken. SPR and SFR are measures of disease burden that avoid the problem of referencing population size when only a small portion of the population is sampled, but could be biased in their own right by the incidence of nonmalarial fevers that would lead people to come to clinics and thus boost the denominator (BSE). %Pf should be a good measure of the relative occurrence of *falciparum *and non-*falciparum *malaria but provides no information on absolute occurrence. Because the standards are high and rigid for labeling of a death as malaria by the NVBDCP (i.e., only when a peripheral blood smear or rapid diagnostic test is positive; even quantitative buffy coat and indirect antibody tests are not recognized), most malaria deaths are not recorded by the surveillance program.

Data were generally collected for districts that were recognized administrative units at time of collection; thus, new districts often have data for only some of the years, and district boundaries change over time within the dataset. We thus aggregated districts as needed to ensure that units were consistent over time, resulting in a final list of 499 districts. We used data from 1995-2005, considering each district in each year as an independent data point for our purposes ("district-years," 5,386 used in this analysis). Data were missing for certain districts in certain years; 103 such district-years were ignored.

#### MDS (Million Death Study)

The MDS gives estimates of cause-specific death rates throughout India, and we used it here to generate estimates of district-year-specific malaria mortality as an external check on the validity of our indices from the NVBDCP. The study was conducted in 1.1 million homes in 6,671 small areas chosen from all parts of India (about 1,000 persons per area) to be representative at the state level. The Sample Registration System was established by the Registrar General of India to monitor all births and deaths in these areas[6,7]. Each home in which a death had been recorded between 2001 and 2003 was visited by one of 900 nonmedical field workers, and the underlying causes of all deaths were sought by verbal autopsy (a structured investigation of events leading to the death conducted by at least two trained physicians) [8-10]. Details of the methods, quality-control checks, and validation results have been reported previously[8,10-12].

For the purpose of this study, we limited our sample of the MDS to deaths occurring at ages 1-69, when misdiagnosis is less problematic and when the bulk of malaria mortality occurs. For each district-year (2001-2003), we calculated the percentage of total deaths in this age range that were attributable to malaria based on the verbal autopsy results. As a check, we also included percentage of deaths due to fever of unknown origin. For some analyses, we included only district-years when there was at least one malaria death.

#### DLHS (District-level Household Survey)

The DLHS is an India-wide, door-to-door household survey that contains questions about whether household members have suffered from malaria recently. Full details of the methodology are available at the Web site of the International Institute for Population Sciences http://www.rchiips.org/ and publications therein[13,14]. We used it here as a way to estimate district-specific malaria morbidity in the years of the survey as an external check on our NVBDCP indices. The DLHS was conducted in three rounds - in 1998-99, 2002-2003, and 2005-06 - but we use data from only the first two rounds. Each round had hundreds of questions, one of which was whether any member of the household had suffered from malaria in the past three months (round 1) or past two weeks (round 2). If the answer was yes, data were collected on the age and sex of the people with malaria and whether they received treatment, for up to five people per household (round 1) or all with malaria (round 2). By combining this with the number of members in the household, we can generate estimates of morbidity as the number of individuals in a district who suffered from malaria in the specified period divided by total individuals in the district. The two rounds provide somewhat different measures of morbidity, but because they are internally consistent across India, they can each be used to validate the NVBDCP indices. Our DLHS 1 sample included 3.2 million individuals, with a mean ± SD of 6380 ± 1680 individuals per district. Our DLHS 2 sample covered 3.5 million individuals, with 5860 ± 760 individuals per district. Thus, even at relatively low malaria levels, our district morbidity estimates should be fairly robust.

### Statistics

**Figure 1:**
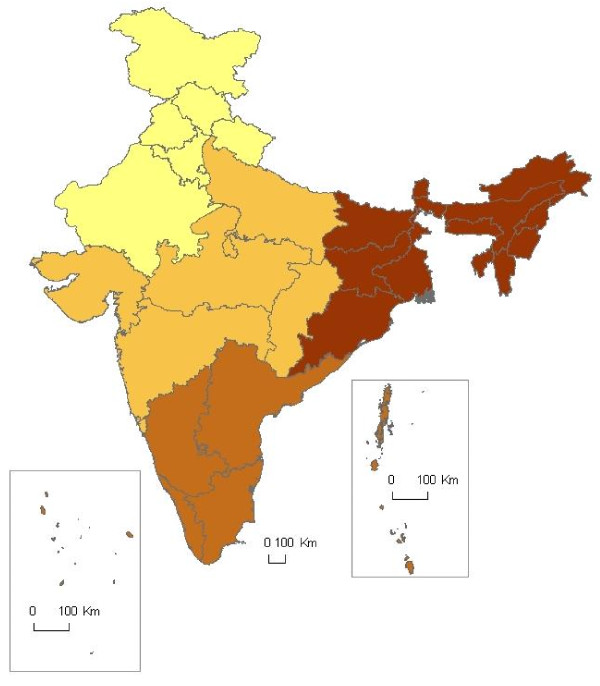
**Map of state groupings used to generate regional analyses**. Grouping of Indian states into regions of north, central, south, and northeast, for the purpose of comparing malaria indices generated in different regions to see if they agree.

All analyses were conducted in R, v. 2.8.0. Data were transformed for normality and standardized by subtracting the mean and dividing by the standard deviation as described in Additional file 1. (A summary of principal components analysis is also provided in Additional file 1.) PCA was run using the princomp function in R. We attempted to generate a general malaria index (SIMS) as the first PCA axis of the analyses, and also pursued but ultimately rejected the idea of an additional *falciparum*-specific index that might better predict mortality. We ran the analyses on 51 subsets of the data: three subsets of the variables for the SIMS and one for the *falciparum *index, and 13 subsets of the observations, in most possible combinations. (The *falciparum*-only measures could not be run on the full dataset due to excessive zeroes). The variable subsets run were: (A) all variables; (B) excluding API and AFI, which should be redundant if ABER, SPR, and SFR are included, and which are hard to interpret given the variation in ABER; (C) excluding API, AFI, and MR, which can be problematic not only because of the large number of zeroes but because in most nonzero districts there was only one death; and (F), the three *falciparum *measures: %Pf, AFI, and SFR. The 13 subsets of observations included: (1) all observations (district-years); (2) district-years with at least one *falciparum *positive slide (SFR>0); (3) district-years with at least one death; (4) district-years with at least 15% of positive slides *falciparum*-positive (%Pf>15); (5) 1995-1996 only; (6) 1997-1998 only; (7) 1999-2000 only; (8) 2001-2002 only; (9) 2003-2005 only; (10) only the northern states of Jammu and Kashmir, Himachal Pradesh, Uttaranchal, Punjab, Haryana, and Rajasthan, as well as Chandigarh and Delhi; (11) only the northeastern states of Orissa, Bihar, Jharkhand, Sikkim, West Bengal, Assam, Tripura, Manipur, Mizoram, Meghalaya, Nagaland, and Arunachal Pradesh; (12) only the central states of Uttar Pradesh, Gujarat, Madhya Pradesh, Chhattisgarh, Maharashtra, and Goa, as well as Daman and Diu and Dadra and Nagar Haveli; (13) only the southern states of Karnataka, Andhra Pradesh, Tamil Nadu, and Kerala as well as Pondicherry, Lakshadweep, and the Andaman and Nicobar Islands. Thus, subsets 2-4 are based on malaria severity, 5-9 are based on time, and 10-13 are based on geography. The geographic divisions are those used in the National Family Health Survey[15], except that eastern and northeastern states are pooled here, as are central and western states (Figure 1). The highest malaria areas in India are almost all in our block of northeastern states. We refer to the analyses based on the numbers and letters described above; for example, B2 would be excluding API and AFI, run on district-years with at least one *falciparum*-positive slide. The *falciparum*-only PCAs were run on the above subsets after excluding all district-years with no *falciparum *recorded. We ran additional validation analyses that are presented in Additional file 1, including using analyses of the raw variables (BSE, PV, PF + mixed, and deaths), and comparing heterogeneity of correlations between the seven indices across the subsets used for PCA validation.

For each of the above PCA analyses, we expected the first PCA axis, which explains the most variation, to be the one of interest. Thus, after assessing the variance that the first PCA axis explained and confirming that this was much higher than for any of the other PCA axes, we took the component loadings for this axis from each of the 51 PCA analyses and used them to calculate PCA scores for the whole dataset. This resulted in 51 new variables, potential indices generated from the subsets of the data. For example, the loadings for the first axis calculated with only the 1995-1996 district-years were used to create a variable from the whole dataset, including all district-years. Comparison between this and similar indices created from the PCAs run on other years allowed assessment of whether the index had changed over time or was stable. We assessed this by calculating the correlations among these 51 axes and with the seven original variables. This was also used to choose a best axis for the Summary Index of Malaria Surveillance based on strength of correlations with the original variables and comprehensiveness. For external validation, we calculated the correlation between MDS-recorded deaths and this axis in the years 2001-2003, and between DLHS-recorded morbidity and this axis in the years 1998-1999 and again for 2002-2003.

## Results

For the 51 PCAs we ran, the variance explained by the first axis was between 49% and 89% (Additional file 2). As predicted, all measures loaded in the same direction on the first axis of each PCA. Even on mutually exclusive portions of the dataset (e.g., Additional file 2, columns 5-9), the variable loadings were nearly identical, suggesting substantial stability of the relationships. The correlations of the indices over these subsets were more variable (see Additional file 1). Additional axes had minimal variance explained and inconsistent loadings, so we retained only the first axis in all analyses. This means that there was no second axis needed to explain relative abundance of *falciparum *and *vivax*; in other words, *falciparum*-to-*vivax *ratio tracks overall malaria levels, as also seen from correlations of the base indices (%PF with API: *r *= 0.51, *p *< 0.0001; %PF with SPR: *r *= 0.49, *p *< 0.0001).

**Figure 2:**
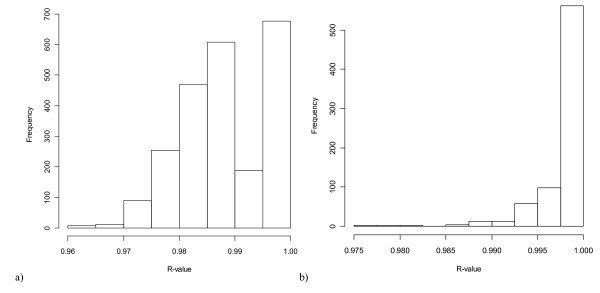
**Histogram of Pearson correlation coefficients among 51 potential indices**. Histogram of Pearson correlation coefficients among the 51 alternative malaria indices generated from subsets of our data (geographic, temporal, or based on malaria levels). The indices are generated from principal component analyses run on the subsets by taking the loadings produced in the analyses and applying them to the full dataset. a) All correlations; b) only correlations among indices generated with the same variable subsets.

**Figure 3:**
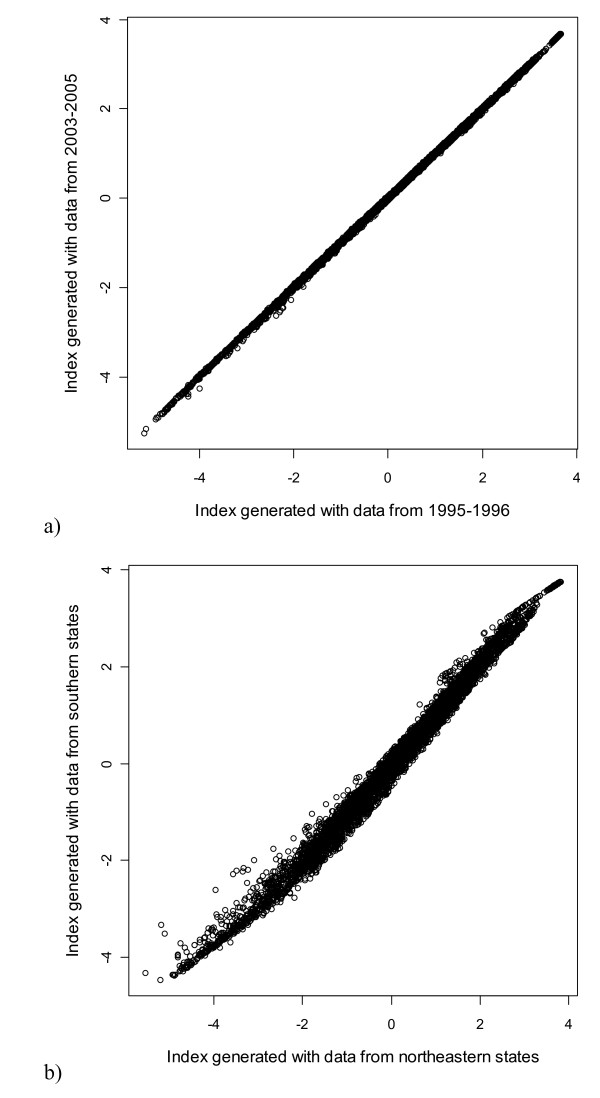
**Correlations between two malaria indices generated from mutually exclusive portions of the dataset**. Loadings from two separate principal components analyses using the measures %Pf, ABER, SPR, SFR, and MR were applied to the full dataset to generate the indices: a) 1995-1996 vs. 2003-2005; b) northeastern states (high malaria) vs. southern states (low malaria).

We generated 51 potential malaria indices for the full dataset based on the loadings from the 51 PCAs. These indices were all tightly correlated with each other - within a given set of variables (A, B, C, or F), correlations were nearly always greater than r = 0.99; across variable sets, the smallest correlation was still r = 0.94 (Figure 2). For all correlations, p < 0.00001. Again, this was true on indices generated from mutually exclusive subsets of the data (e.g. Figure 3). This indicates that all of our indices are essentially equivalent and would serve as a good proxy for malaria trends. We selected B1 for the SIMS based on its inclusion of all observations possible and maximal correlations with the original measures. B1 is also preferred because it excludes API and AFI, which are composite indices generated from the same information contained in the ABER, SPR, and SFR (i.e., API = SPR/ABER and AFI = SFR/ABER). The mathematical properties of a principal components analysis on such redundant but nonlinear information are not well understood, and we felt it safer to exclude these measures in the absence of any other strong justification. Based on the axis loadings, the log- or square-root transformations, the standard normal transformations, and scaling considerations, the following formula can be applied to malaria data to calculate the SIMS:

SPR, SFR, and MR include a minor correction factor of 0.001 because they are frequently zero, and log(0) = -∞; see Additional file 1. Each of the five indices is then log or square-root transformed for normality. They are each turned into standard normal random variables by subtracting the mean and dividing by the standard deviation, then multiplied by the appropriate axis loadings from PCA B1. The additional adjustments are for the purpose of scaling and are described in detail below. We provide a spreadsheet online that can be used to calculate the SIMS either from raw data or from the existing indices (Additional file 3).

**Figure 4:**
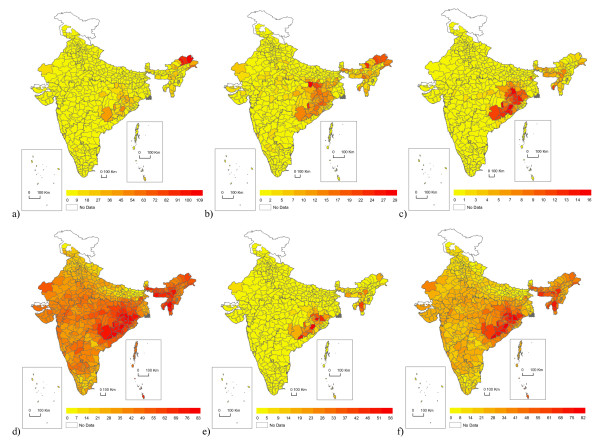
**Maps of SIMS and other malaria indices by district in India, averaged 1995-2005**. a) API (annual parasite index); b) SPR (slide positivity rate); c) SFR (slide *falciparum *rate); d) a normally distributed version of the SIMS (Summary Index of Malaria Surveillance); e) a fully exponentiated version of the SIMS; and f) the final SIMS. Scales differ across measures, but colors are divided into 12 equal classes comprising the full range of values for each measure and ensuring visual comparability across a) through f) for inferring the distribution. Note that the exponentiated SIMS matches the API, SPR, and SFR, but fails to distinguish well among districts at the lower end of the spectrum. The normally distributed SIMS fails to convey how much worse the problem is in high-malaria districts relative to most districts.

The original PCA axis was normally distributed; however, since actual malaria burden (as measured by API, SPR, and SFR, for example) is closer to log-normally distributed, this would be misleading for the index (Figures 4a, b, c, and 4d). Unfortunately, exponentiating the raw PCA axis gives a distribution that fails to highlight relevant variation at the lower end of the spectrum: 63% of district-years are in the lowest 1% of the range, and 89% are in the lowest 5% (Figure 4e). The purpose of the index is to facilitate comparisons over space and time, and it thus must highlight variation throughout the spectrum: the most severe districts should appear exceptional (as they are), but distinctions among less severe districts must also be possible. Thus, we took the exponent of 1/5 of the raw PCA axis - an adjustment that gives a distribution intermediate to normal and log-normal, allowing some sense of the actual range of severity while preserving distinctions on the lower end of the spectrum.

**Figure 5:**
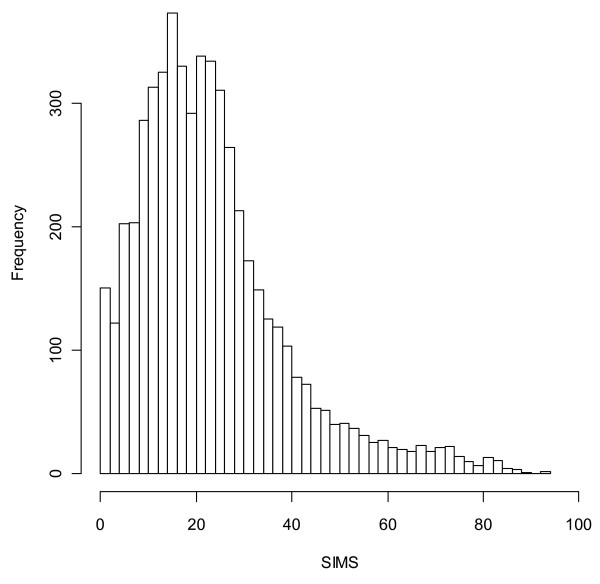
**Histogram of values of the Summary Index of Malaria Surveillance (SIMS) taken by the district-years in our sample**. Mean is 23.3, median is 20.6, and mode is approximately 18.

The final task was to put this measure on a scale of 0 to 100, leaving room for values that might lie outside the range we observed. We would have liked 0 and 100 to be precise theoretical maxima and minima, but this is not possible if we wish to preserve the roughly realistic semi-exponential distribution. The choice of how much additional range to leave was arbitrary. To scale our observed points from 0 to 100, we would subtract the minimum (0.483) and divide by the range (2.31) of the unscaled index, then multiply by 100. We chose instead to use 0.48 and 2.5 respectively, resulting in our observed values ranging between 0.12 and 93. This decision was made on the assumption that some of our districts had essentially no malaria (0.12 is very close to 0.00), but that there was more room at the upper end for transmission levels higher than we had observed. With these adjustments, the SIMS is easily interpretable (Figures 4f and 5).

**Figure 6:**
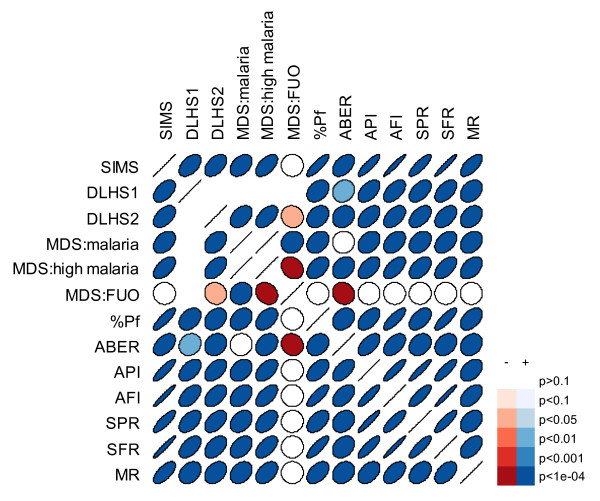
**Correlations between various measures of malaria by district-year in India**. Ellipses indicate Pearson correlation coefficients, with narrower ellipses showing stronger correlations. Right-slanted and blue indicate positive correlations, left-slanted and red indicate negative. SIMS = Summary Index of Malaria Surveillance; DLHS = morbidity from the District-level Household Survey, rounds 1 (1998-99) and 2 (2002-03); MDS = fever mortality from the Million Death Study, either malaria mortality ("malaria"), malaria mortality in only districts that had at least one malaria death ("high malaria") or fever of unknown origin ("FUO")); %Pf = percent of positive slides that are *falciparum*-positive; ABER = annual blood examination rate; API = annual parasite index; AFI = annual *falciparum *index; SPR = slide positivity rate; SFR = slide *falciparum *rate; MR = deaths/population.

For comparisons of correlations among variables, all were transformed as necessary to achieve approximately normal distributions. The SIMS correlated with percent of deaths attributable to malaria by district-year according to MDS and with self-reported malaria morbidity according to the DLHS (Figure 6, Additional file 4). This was better than most of the individual measures, though SFR was at least as closely associated with MDS deaths, and API and AFI were at least as well associated with DLHS morbidity. However, the SIMS was clearly unassociated with MDS deaths due to fever of unknown origin, providing evidence both that the SIMS is picking up the appropriate trends and that the MDS verbal autopsy methodology does not underestimate malaria due to vague diagnoses.

## Discussion

We have shown that principal components analysis can be used to generate a robust index of malaria incidence, the SIMS, based on summary measures of blood data collected by district and year throughout India. This index will provide a simpler way to quantify and interpret temporal and geographic variation in malaria in India because multiple measures need not be considered simultaneously. In some cases, the individual measures will still be more appropriate - for example, to compare relative trends of *falciparum *and *vivax *malaria. However, in most cases, a single, more comprehensive measure will be preferable. Even for prediction of mortality according to the MDS, the SIMS fares as well as the *falciparum*-specific measures.

It is possible to generate a summary measure (valid or not) from almost any set of variables, so a rigorous standard must be used to validate any such summary and to clarify its interpretation[16]. Here, there are multiple strands of evidence suggesting that the SIMS is valid and has a clear interpretation:

(1) All of our individual variables load onto the SIMS in the same direction, as would be predicted.

(2) The SIMS explains a substantial portion of the overall variation, 58%.

(3) The results were essentially unchanged after omitting observations with zeroes from the dataset.

(4) Results were almost exactly identical even when the analysis was run on mutually exclusive subsets of the data from different years or geographic regions.

(5) Results were quite similar even when using different combinations of the indicator variables.

(6) The SIMS correlates as well or better than the individual measures with external measures of malaria mortality and morbidity.

It is unusual that summary measures generated from mutually exclusive subsets of the data would correlate so well with each other, and this result has some implications. First, SIMS is likely to be stable over time and space. The component loadings generated in this study should be applicable to new data generated in the future, without the necessity to run new PCA for the new dataset and without the potential for conflicting results. Second, the measurement error appears to be relatively consistent over time. If measurement error varied, the loadings would likely become unstable, too.

Third, it seems that malaria trends, at least as measured by the NVBDCP, are a real phenomenon that can be described adequately with a single dimension. More specifically, there appears to be a stable pattern of stages of severity in malaria transmission, as evidenced by the ability of one axis to describe both species ratio and overall abundance. At the lowest levels, malaria is essentially absent. Then *vivax *comes in at low levels. Only when *vivax *reaches moderate to high levels does *falciparum *appear, and at the highest levels, *falciparum *is usually much more common than *vivax*. This could be an example of ecological niche partitioning between these species, with one favored by conditions of low, stable transmission and the other favored by high transmission (i.e., high mosquito densities and bite rates) and mortality rates[17,18]. If this is the case, it might bode poorly for the hope that *falciparum *will eventually evolve lower virulence as that niche is already occupied by a competitor species [19-21]. However, there are many other potential explanations for this pattern. It is possible that the species occupy different niches without competing, or that there are sampling biases against detecting *vivax *in high *falciparum *areas, for example.

The main caution in the use of the SIMS going forward is that the NVBDCP has been improving its data collection methodology, including use of rapid diagnostic tests and computerized data entry (GPS Dhillon, personal communication). This is unquestionably a laudable development, but may inadvertently cause problems with comparability of data over time and space, and it is possible that future values of the SIMS will not be fully comparable to past ones. However, it is also possible that past variation in sampling accuracy is also large, and that the SIMS is successfully detecting signal among the noise in ways that will be unaffected by these changes in data collection methodology. For example, NVBDCP surveillance data rely only on the public health sector, and private facilities and persons who do not seek care are not covered; thus, past surveillance coverage has presumably varied with time and space. Despite this, the SIMS has so far proven stable. Whether this continues to be the case will have to be validated in the future. If the future SIMS proves incomparable to past SIMS, existing surveillance measures such as API should be even harder to compare because incomparabilities would arise due to changes in surveillance methodology affecting all indices, and the SIMS has some ability to buffer these changes by extracting signal from noise. Even if past and future SIMS cannot be directly compared, it is likely that the SIMS as shown here would be a stable measure for comparison within any dataset collected with consistent methodology.

It will also be important to establish the relationship between SIMS (essentially a measure of transmission intensity) and direct measures of burden such as prevalence, incidence, and mortality. It may or may not be possible to directly predict these burden measures from the SIMS after sufficient validation. Regardless, the SIMS should serve as a replacement for the existing indices in many situations, particularly in statistical and data presentation applications where a single measure is preferable to many. We have provided a table of approximations of relationships between the SIMS and the seven existing indices that can be used by policymakers to decide what levels of SIMS would correspond to existing thresholds for policy decisions (Table 2). The SIMS should be sufficient without conversion, but many users may wish to see such a table of equivalences until they are more familiar with the SIMS.

**Table 2:** Relationship between the SIMS and existing indices

SIMS	API	AFI	SPR	SFR	%PF	ABER	MR
0	0.0	0.0	0.0	0.0	0.0	3.0	0.0
1	0.11	0.03	0.16	0.03	0.5	3.4	0.04
10	0.30	0.10	0.37	0.11	3.3	6.3	0.05
20	0.69	0.30	0.76	0.31	15.1	9.3	0.06
40	2.2	1.3	2.0	1.3	45.6	14.5	0.08
60	4.9	3.8	4.1	3.4	77.1	18.9	0.09
80	9.3	8.6	7.0	7.0	100.0	22.6	0.11
100	15.5	15.5	10.9	10.9	100.0	26.0	0.12

Approximate correspondence of SIMS values to values of existing indices, based on a combination of linear models and theoretical maxima and minima. SIMS: Summary Index of Malaria Surveillance; API = annual parasite index; AFI = annual *falciparum *index; SPR = slide positivity rate; SFR= slide *falciparum *rate; %Pf = percent of positive slides that are *falciparum*-positive; ABER = annual blood examination rate; MR = deaths/population

The SIMS described here should help improve the clarity of malaria surveillance in India and perhaps elsewhere. It is designed for easy use and interpretation by people with no statistical background. In particular, when looking at maps and graphical representations of malaria distribution, it will help to have a single measure rather than several of uncertain interpretation. The strong support we get for a stable measure suggests that it would be worthwhile to pursue similar indices in other settings where different measures of malaria have been collected[22]. Within India, this measure may serve as an analytical tool for researchers assessing the progress of control and eradication programs, and also perhaps as a clear benchmark for holding local authorities accountable for progress. Internationally, it seems likely that similar principles will facilitate the ability to generate a single standard index for malaria transmission intensity.

## Conclusions

We have demonstrated that principal components analysis can be used to construct a single measure, the SIMS, that summarizes most relevant variation in malaria surveillance measures across time and space. It can be interpreted as a relative measure of transmission intensity. Species abundance tracks overall levels, meaning that a separate measure is not needed. The SIMS is robust over time and space - alternate versions calculated from subsets of the data did not differ noticeably. We have provided a spreadsheet calculator, ensuring that even field workers with no mathematical background can accurately use the measure. We expect that the SIMS will simplify and improve malaria surveillance in India, and that similar measures should be applicable in other settings as well.

## Competing interests

The authors declare that they have no competing interests.

## Authors' contributions

AAC designed and carried out the analysis and wrote the manuscript. ND, RJM, VPS, and PJ provided and helped interpret data, helped structure and interpret the analyses, and edited the manuscript. PSR integrated the data, produced the maps, and edited the manuscript. All authors read and approved the final manuscript.

## Supplementary Material

Additional file 1 Data preparation, Additional validation, Methodological Discussion, and Introduction to Principal Components Analysis. MS Word document, SIMS Appendix A_rev.doc. Click here for file

Additional file 2 Variance explained and loadings of raw malaria measures on the first principal component axis, run with various subsets of the data, SIMS_PHM_Table_S1.xls. Click here for file

Additional file 3 SIMS calculator in an MS Excel spreadsheet, SIMS calculator.xls. Click here for file

Additional file 4 Correlations among index measures and external measures of malaria morbidity and mortality. MS Excel Spreadsheet, SIMS_PHM_Table_S2.xls. Click here for file
