# Supplementary material for: The Summary Index of Malaria Surveillance (SIMS): a stable index of malaria within India
Source: Popul Health Metr. 2010 Feb 11;8:1. doi: 10.1186/1478-7954-8-1 (PMC2847540; doi:10.1186/1478-7954-8-1)
Supplement: Additional file 1 — Data preparation, Additional validation, Methodological Discussion, and Introduction to Principal Components Analysis. MS Word document, SIMS Appendix A_rev.doc. [file 1478-7954-8-1-S1.DOC]

**Additional File 1: Supplementary Methods and Results**

*Data preparation*

We began by re-creating the standard measures from the raw data. In particular, we found that there was inconsistency across district-years as to whether mixed cases (those positive for both *P. falciparum* and *P. vivax*) were included in the numerator of *falciparum* indices. We opted to include them, and recalculated the indices accordingly. This also eliminated several instances in which the original indices were implausibly high (e.g., SFR=242%). We ran all analyses with both the original values and our recalculated values, but the results do not differ qualitatively and we present here only the results based on our recalculations. We calculated mortality ratios (MR) by dividing deaths by the district population.

We transformed each measure to as close as possible to a normal distribution. Normality is hard to assess statistically when sample size is large because even small deviations become significantly non-normal – we were constrained by R to assess normality on a subset of 4999 district-years. We attempted to maximize the W statistic from Shapiro-Wilk tests across several transformations: untransformed, log-transformed, square-root transformed, and inverse. In addition, because there were a fair number of zero-values for the low-malaria districts, we ran the Shapiro-Wilk tests with and without these values for each transformation; when zeroes were included, we added a minimal correction factor to them of 0.001 so that logs could be calculated. Without zeroes, the W-statistics for the chosen transformations are: square-root %Pf (W=0.96); square-root ABER (W=0.94); log-API (W=0.98); log-AFI (W=0.99); log-SPR (W=0.98); log-SFR (W=0.99); log-MR (W=0.98). However, many district-years had no deaths and a fair number had no *falciparum*; in these cases, no transformation can make the data approach normality. We account for this by excluding these districts from some analyses. Before running PCA, we transformed all our variables one more time into standard normal random variables (subtract the mean and divide by the standard deviation) so that they were all on the same scale. This was necessary so that we could cross-apply component loadings from subdivided data sets.

NVBDCP districts are not always precisely the same as official Indian administrative districts. For example, major cities in Tamil Nadu were recorded separately from the rest of their districts. For our purposes, we defined districts as units having separate data for all years in which data were available. For example, the Punjabi districts Moga and Mukatsar were merged with Faridkot in 1995 (the districts hadn’t separated yet), but had separate data for 1996-2005. We thus treat the three as a single district, summing the populations and malaria levels for the later years. In contrast, Krishnagiri district in Tamil Nadu did not officially separate until 2004, but had its data recorded separately for all years, and was thus treated as a separate district. By this method, we had 499 districts.

District population estimates provided by the NVBDCP were often problematic. Ultimately, all population estimates are based on the census, but each state separately compiles its own data, and some states used much finer-scale interpolations of the census data than others. Because the 1991 census estimates are generally much lower than the 2001 estimates due to population growth, coarsely interpolated states appear to have sudden jumps in population around 2001, but do not have equivalent increases in the malaria measures for which population serves as a denominator. However, NVBDCP districts are often not exactly equivalent to census districts, and also occasionally have years for which part of the district is not sampled. In these cases, using census estimates could result in the spurious appearance of a 1-year drop in malaria levels. Because the NVBDCP data contained a number of typographical errors as well, it was not always possible to determine the correct population number. We have taken the approach of using linear interpolations from the 1991 and 2001 censuses in cases where we do not have particular reason (e.g. sudden concomitant shifts in malaria numbers) to think that the NVBDCP population numbers reflect peculiarities of the sampling. In cases where it appears to reflect sampling peculiarities, we either use the original numbers or adjust our interpolations to account for the peculiarities. The process necessitated many judgment calls and cannot be easily replicated; however, data and notations are available upon request. Results with the revised population estimates did not differ much from preliminary analyses.

*Additional validation*

We conducted several additional validation studies in addition to those in the main text. First, we ran PCA on the four key raw variables (BSE, PV, PF+mixed, and Deaths), all divided by population. Because the indices used in the main analysis are not statistically independent, it was felt that it would be important to confirm the results using four statistically independent measures. Each was log-transformed to an approximately normal distribution after assessment of normality of several transformations using Shapiro-Wilk scores. As in the main analysis, the first axis explained the bulk of the variation (53%), and all variables loaded substantially in the same direction on the axis. The axis generated has a fairly strong correlation with the normally distributed version of the SIMS (r=0.79, p<0.0001, Fig. A1), but not as strong as the correlations among alternate versions of the SIMS.

This is probably attributable to the different interpretation needed for a PCA on the raw variables. Had we not divided by population, the main axis would likely have indicated district population, since districts with large populations are likely to collect more slides and find more positives; other axes might also have been important. We obviate this problem by dividing by population, but create a new problem: districts with many slides collected per population are also likely to find more positives per population. The first axis generated here is thus at least partly describing intensity of sampling, as opposed to intensity of malaria transmission. In other words, the raw variables are statistically but not functionally independent, and the interpretation of the analysis must reflect that. On the other hand, the composite variables used in the main analysis, with the exceptions of API and AFI, are not statistically independent, but are functionally independent (or at least could be: that is one thing we are trying to assess). So, while it is good to confirm a broadly consistent result from the analysis of the raw variables, it is difficult to interpret precisely what the minor discrepancies mean.

One of our main findings was that the PCA axis is stable over time and space. We assessed whether this could also be said of the correlations among the indices used to generate the SIMS. We took all 21 pairwise combinations of the seven indices and tested for heterogeneity of the correlations separately across the five temporal and four geographic subsets used in the main analysis. To do this we used *z*-transformations of the correlation coefficients and compared the difference between observed and expected weighted sums of squares to a chi-squared distribution. Across the temporal subsets, 12 of the 21 correlations showed significant heterogeneity (p<0.05); across the geographic subsets, all 21 did (p<0.0001 for all). In other words, the pairwise correlations do change substantially over time and space. We suspect that the reason the PCA appears stable while the individual correlations are not is attributable to summary of information that occurs with the PCA. A pairwise correlation will change if there is a change in the measurement error for either index; PCA, by finding the shared variation, is combining enough different indices that the measurement error of each will not play much into the final result.

Fig. A1 – Correlation of SIMS with first axis of a PCA analysis on four raw variables (BSE, PV, PF+mixed, and Deaths), each divided by population. *r*=0.79, *p*<0.0001.

*Methodological Discussion*

Four of our original measures – SFR, %Pf, AFI, and deaths – had a large number of zeroes. Out of 5386 district-years, 175 had no malaria, 750 had no *falciparum*, and 4054 had no deaths. Zeroes pose a challenge in an analysis like this because they cause the data to become highly non-normally distributed and cannot be transformed. However, our results did not change noticeably when we omitted these data points, giving us some indication of the validity of the measures. From a practical standpoint, it is not necessary to come up with an accurate index for when there is no malaria: there is simply no malaria, and knowing that is enough. The SIMS is designed to measure how severe malaria is when there is malaria present (values of the SIMS under 5 generally indicate no malaria detected). Likewise, it appears that *falciparum* increases as the other indicators become worse, such that areas without *falciparum* are appropriately scored low on the index. When *falciparum* is zero, the index then becomes the sum of the other indices multiplied by their loadings. We thus believe that the SIMS will tend to be generally relevant as long as districts have some malaria, but caution must be used with regard to precise estimates when malaria levels are low or when there is no *falciparum*.

In some situations, individual measures may be preferred, and standard practice has been to accept API as a valid measure only when ABER exceeds 10%. However, we found ABER to be continuously distributed, with a mean of about 10.7% and some 90% of values below 20%. This does not support the arbitrary use of a 10% threshold: 10.1% is not qualitatively different from 9.9%, and many values fall right in this range. ABER is almost never high enough that we should feel confident in extrapolating from it to the entire population. Since API = SPR × ABER, and since ABER may be a measure of fever incidence in the population, we favor use of SPR and ABER but not API as an alternative to the current practice.

*Introduction to principal components analysis*

Principal components analysis (PCA) is a technique used to extract shared variation from a set of variables. For example, imagine we want to measure body size. Height is not a sufficient measure: a skinny person is not as large as a stout person of the same height. But weight is also not sufficient, since a tall skinny person might seem larger or more imposing than a short, stout person of identical weight. Ideally, we want to combine the information from both measures. To do this, we put these variables, along with others such as waist circumference, limb length, finger length, and as many more body-part-size measures as we can imagine into a single PCA. What the PCA does is to calculate a correlation matrix (or a covariance matrix, in some situations) among all the variables. Matrix algebra is then used to find linear combinations of the variables that will create a new set of variables, or “axes.” This new set will have the same number of axes as the variables we originally put into the analysis, but will differ in a very important way. The first axis in the set will explain as much of the total variance in all our variables as can possibly be explained with a single variable. (If our original variables are all highly correlated, this will be a very large percent). For example, our first axis might be height×0.5+weight×0.35+waist circumfrence×0.1, etc., if this combination were the one that explained as much variance as possible. After generating the first axis, a second axis is calculated that is orthogonal (perpendicular) to the first, and also explaining as much of the remaining variance as possible. This process is repeated until the number of PCA axes is equal to the number of original variables, when there will be no remaining variance left to explain.

In our body size example, the first axis we generate will likely represent large versus small people. Large people will be taller, heavier, have larger waists and longer limbs, etc. Our second axis is likely to represent relative stoutness versus slenderness. If we included many measures, we might have other axes later in the list that would represent, say, both finger pudginess and having a bulbous nose (which might be associated). The point is that our original measures were not independent – people who are large by one measure tend to be large by others – but that the ways we would really like to characterize body size and shape are not easily understood from the things we can measure directly. By using PCA, we can take the things which we can measure, and which are correlated but not identical, and use them to identify the underlying patterns that we want to get at. Of course, there is no guarantee that PCA will give us a meaningful result – it is possible that body size variation will not follow the patterns we expect, and the axes may not be readily interpretable. In this case, PCA cannot be used, but the lack of a clear interpretation can itself be informative.

There are several important parts to the output of PCA. The first is the variance explained. If we put 5 variables into the PCA, we do not want the five new axes to each explain 20% of the variation – if so, we have not improved over our original measures. We would much rather see the five axes explain 80%, 15%, 3%, 1.5%, and 0.5% of the variation, since it means we can use the first one or two axes and forget about the rest. The second important part of the output is the component loadings. For each axis, this is a list of weights assigned to each of the original variables in order to create the linear combination for the axis. For example, these were 0.5, 0.35, and 0.1 for height, weight, and circumference above. If the loadings for a given axis are all positive (or all negative), it means that the original variables all have the same direction of association with the axis – positive correlations, for example. If some are positive and some are negative, however, that axis represents the variance explained by the tendency of those variables to be negatively correlated. For example, if the second axis of our body size example represents relative slenderness versus stoutness, we would expect height and waist circumference to be negatively correlated: a very slender person should be taller but with a narrower waist, and a very stout person should be shorter but with a larger waist. So one would load positively and one would load negatively on the second axis. (It doesn’t really matter which is positive and which is negative – if they are inverted, then the interpretation of the axis shifts from high value=slender to high value=stout, but is otherwise the same.

The last important part of the output of a PCA is the scores. Scores are generated by using the component loadings to calculate a value on each axis for each of the original observations in the data set. In our example, each of the people in our data set would get a “size” score on axis 1, a “slenderness” score on axis 2, etc. These scores could then be used as a variable or index in subsequent analyses of that data set. If we really wanted to measure size, this would be a much better way to do it than to try to make sense out of all our original variables simultaneously, since there are fewer variables to consider and we have extracted all the relevant size variation from our original set of variables. The caveat is that we must be careful that our axis really represents size well: does it explain a lot of the variance in our original variables? Do all the original variables load on that axis in the same direction? Do the variables we expect to load more strongly (i.e. weight and height, as opposed to nose breadth) actually do so? Does our new axis correlate with external measures that we might expect it to (say, survey results about how annoyed people are about the size of airplane seats)?

Lastly, we might wish to know how general our results are. Is the size axis we came up with very general, or does it apply only to the people in our original data set? Maybe the body proportions associated with being large are very different for African Pygmies than for Norwegians, and the loadings generated from an analysis of pygmies would not apply well to Norwegians. Or maybe the proportions are different for women and men. They almost certainly differ for children and adults, since head size is a much larger proportion of body size in children. If the results do not differ across such sub-groups, then we can apply our loadings to size measurements from any population and get a reasonable estimate of size, but if they do differ, we must generate our size axes separately for the different groups and must be very careful about comparing these axes, since they are apples and oranges.
